# A multidrug-resistant organism tracking and alert system is associated with lower MRSA and CP-CRE infection rates at Veterans Affairs Medical Centers

**DOI:** 10.1017/ash.2026.10787

**Published:** 2026-07-14

**Authors:** Amanda Vivo, Cara Ray, Tarek El Ali, Geneva M. Wilson, Reside Jacob, Makoto M. Jones, Natalie R. Hicks, Christopher D. Pfeiffer, Charlesnika T. Evans

**Affiliations:** 1 VA Center of Innovation for Complex Chronic Healthcare, Hines VA Hospital, Hines, Illinois, USA; 2 Northwestern University Feinberg School of Medicine, USA; 3 Center for Health Services and Outcomes Research, Department of Preventive Medicine, Northwestern University, Chicago, USA; 4 VA Salt Lake City Health Care System, USA; 5 Division of Epidemiology, Department of Internal Medicine, University of Utah School of Medicine, Salt Lake City, USA; 6 National Infectious Diseases Service, Veterans Health Administration, US Department of Veterans Affairs, Washington, DC, USA; 7 Portland VA Health Care System, USA; 8 Oregon Health & Sciences University, USA

## Abstract

This study investigates Veterans Affairs Bug Alert (VABA) usage on methicillin-resistant *Staphylococcus aureus* (MRSA) and carbapenemase-producing carbapenem-resistant Enterobacterales (CP-CRE) infection rates in VA facilities. Significant association between increased VABA use and decreased MRSA and CP-CRE infection rates supports VABA as part of infection control strategies.

## Introduction

Multidrug-resistant organisms (MDROs) significantly threaten public health, prolonging hospital stays, and increasing treatment complications and mortality rates.^1,2^ MDROs such as methicillin-resistant *Staphylococcus aureus* (MRSA) and carbapenem-resistant Enterobacterales (CRE) are serious and urgent threats, as designated by the Centers for Disease Control and Prevention (CDC) and are frequently transmitted in healthcare settings.^3–5^ Carbapenemase-producing carbapenem-resistant Enterobacterales (CP-CRE) is a subset of CRE emerging globally with a higher risk of transmission and mortality.^6^

Addressing MDROs demands a multifaceted approach encompassing judicious antimicrobial use, robust stewardship programs, and effective infection control measures. VA Bug Alert (VABA) was designed by the Veterans Health Administration as an infection control tool to reduce MDRO transmission. VABA notifies infection prevention personnel at VA Medical Centers (VAMCs) when a patient with a history of colonization or infection with an MDRO is admitted to acute or long-term care in their facility.^7^ Using VABA allows healthcare providers to quickly identify and flag high-priority MDROs including but not limited to MRSA and CP-CRE. This proactive approach may enable timely placement of MDRO-positive patients into appropriate transmission-based precautions, effectively disrupting the chain of transmission and impeding further spread.

The objective of this analysis was to investigate the association between VABA use and healthcare-associated MRSA and CP-CRE infection rates in VAMC acute care and intensive care units.

## Methods

### Research design and data sources

This was an ecological study evaluating VABA utilization, infection rates, and facility characteristics using VA administrative records from May 1, 2022 – December 22, 2023. Facility complexity was based on patient characteristics, clinical programs, and teaching programs and categorized as high, medium, or low complexity. Geographic region was based on U.S. Census Bureau categories. VAMC-level healthcare-associated infection (HAI) MRSA and CP-CRE rates and bed days of care (BDOC) were obtained from the VA Inpatient Evaluation Center. VABA utilization was defined as the number of days at least one VAMC staff member viewed currently admitted or recently discharged patients with VABA-eligible MDROs in VABA. MRSA and CP-CRE HAI was defined as infection that developed on or after the third calendar day of admission to an inpatient facility, following the CDC National Healthcare Safety Network definition for HAIs.^8^ MRSA and CP-CRE HAI rates were defined as number of MRSA HAIs or CP-CRE HAIs per 1,000 BDOC.

### Statistical analysis

Fisher’s exact test and Kruskal–Wallis test were used to compare facility characteristics across facility complexity. The correlation between days of VABA utilization and complexity-adjusted MRSA HAI and CP-CRE HAI rates were calculated using Pearson’s correlation coefficient. The rate of MRSA HAI and CP-CRE HAI at each facility had two distinct distributions with a concentration of observations at 0 and the rest of the observations following a gamma distribution. Thus, a two-part finite mixture model (including gamma-distributed and constant-rate components), was used to analyze the relationship between MRSA HAI and CP-CRE HAI rates and days of VABA use. The model adjusted for facility complexity level, and model fit was determined based on Akaike Information Criterion (AIC), Bayesian Information Criterion (BIC), and Pearson statistics. Analyses were performed using the FMM procedure from SAS version 9.4 (SAS Institute) and R version 4.5.2.

## Results

One hundred and eight VA facilities were included in the analysis. Most facilities were high-complexity (73.1%), followed by medium (14.8%) and low (12.0%). Nearly all were in urban areas (90.1%). Regional distribution across the US shows that 40.7% of facilities are in the South, 21.3% in the West, 19.4% in the Midwest, and 18.5% in the Northeast. The mean number of days VABA was utilized during the study period was 19, 25, and 56 days in lo w-, medium-, and high-complexity facilities, respectively, with an overall range of 0 to 484 days. The average MRSA infection rate across VAMCs was 0.11 per 1,000 BDOC, with the highest rate observed at 0.97 per 1,000 patient BDOC and the lowest at zero. The average CP-CRE infection rate was 0.01 per 1,000 patient BDOC, with individual VAMCs ranging from zero to 0.27 per 1,000 BDOC (Table 1).

**Table 1.** Facility characteristics Table 1 long description.

Characteristic	N	Low complexity N = 13	Medium complexity N = 16	High complexity N = 79	*P*-value
**Rurality**	108				<.001
Rural		5/13 (38%)	4/16 (25%)	1/79 (1.3%)	
Urban		8/13 (62%)	12/16 (75%)	78/79 (99%)	
**US Region**	108				.081
Midwest		4/13 (31%)	3/16 (19%)	14/79 (18%)	
Northeast		3/13 (23%)	5/16 (31%)	12/79 (15%)	
South		1/13 (7.7%)	6/16 (38%)	37/79 (47%)	
West		5/13 (38%)	2/16 (13%)	16/79 (20%)	
**Days of VABA use**	108				.3
Median (25%;75%)		13 (6;24)	20 (4;35)	22 (5;69)	
Min-Max		2–87	0–122	0–484	
Mean (SD)		19 (22)	25 (30)	56 (83)	
**Facility infection rate (CP-CRE)**	108				.2
Median (25%;75%)		0.00 (0.00;0.00)	0.00 (0.00;0.00)	0.00 (0.00;0.00)	
Min-Max		0.00–0.00	0.00–0.27	0.00–0.25	
Mean (SD)		0.00 (0.00)	0.02 (0.07)	0.01 (0.04)	
**Inpatients (CP-CRE)**	108				<.001
Median (25%;75%)		21,756 (19,011;25,614)	33,305 (26,472;38,832)	59,015 (44,519;82,883)	
Min-Max		11,286–32,884	12,300–55,635	20,564–125,373	
**Facility infection rate (MRSA)**	108				<.001
Median (25%;75%)		0.00 (0.00;0.00)	0.00 (0.00;0.02)	0.10 (0.05;0.17)	
Minimum-maximum		0.00–0.56	0.00–0.85	0.00–0.97	
Mean (SD)		0.08 (0.19)	0.08 (0.21)	0.12 (0.13)	
**Inpatients (MRSA)**	108				<.001
Median (25%;75%)		21,756 (19,011;25,614)	33,305 (26,772;38,469)	59,015 (44,880;81,527)	
Minimum-maximum		11,286–32,884	12,300–55,635	20,564–125,373	

Note: VABA, Veterans Affairs Bug Alert; CP-CRE, carbapenemase-producing carbapenem-resistant Enterobacterales; MSRA, methicillin-resistant Staphylococcus aureus.

**Figure 1.**
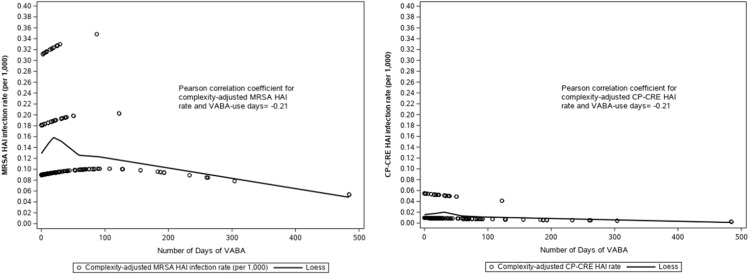
Methicillin-resistant *Staphylococcus aureus* and carbapenemase-producing carbapenem-resistant enterobacterales hospital acquired infection rates by number of days of VA Bug Alert use. (a) Methicillin-resistant *Staphylococcus aureus* hospital-acquired infection rates by number of days of VA Bug Alert use. (b) Carbapenemase-producing carbapenem-resistant Enterobacterales hospital-acquired infection rates by number of days of VA Bug Alert Use. *Note.* (a) Akaike Information Criterion—11.26. Bayesian Information Criterion—7.51. Note. (b) Akaike Information Criterion—45.65. Bayesian Information Criterion—61.74.

Pearson correlation coefficients suggested a weak negative linear relationship between the days of VABA use and infection rates (Figure 1). There was no significant association between MRSA HAI rates and the days of VABA utilization in unadjusted analyses. In the gamma component, after adjusting for facility complexity, for each increase in day of VABA use there was a decrease in MRSA infection rate of 0.002 (95% CI 0.0004–0.004, *P*-value = .01). In other words, a 10 day increase in VABA use was associated with a 2.2% decrease in mean MRSA infections (95% CI 0.4%–3.9%, *P*-value = .01). There was no significant association between CP-CRE HAI rates and days of VABA utilization in unadjusted analyses. However, after adjusting for facility complexity, the gamma component showed that each additional day of VABA use was associated with 0.995 decrease in infection rate (95% CI 0.990–0.999, *P*-value = .04). Thus, for each 10 day increase in VABA use, there was an associated 5% lower CP-CRE infection rate (95% CI of 0.1% to 9% decrease). In a sensitivity analysis removing the outlier of 484 days of VABA use, the relationship between VABA use and MRSA and CP-CRE HAI rates had the same direction but a lesser magnitude. After assessing model fit statistics, all observations were included in the final model.

## Discussion

The findings of our study provide valuable insights into the relationship between the use of a tracking and alert system, VABA, and MRSA and CP-CRE HAI rates within VAMCs. The analysis revealed a significant association between the number of days of VABA use and decreased MRSA HAI rates and a borderline significant association between number of days of VABA use and decreased CP-CRE HAI rates after adjusting for facility complexity. While the study design does not allow for the conclusion of a causal relationship, the observed association between VABA use and reduced MRSA and CP-CRE HAI rates underscores the potential of this tool as part of comprehensive infection control strategies in VAMCs.

These results have important implications for infection control practices within VAMCs and other healthcare settings. Electronic health records can facilitate the automated collection of surveillance data and support infection control interventions at the point of care, enabling healthcare practitioners to implement contact precautions more quickly to control the spread of infections.^9,10^

This analysis had several limitations. Firstly, the absence of patient-level data restricts the ability to assess individual patient characteristics that may influence MRSA and CP-CRE transmission and infection rates. Additionally, ecologic data limits the ability to establish causality and temporality between VABA utilization and MRSA and CP-CRE HAI rates. Furthermore, the evolving policy landscape within VAMCs, particularly regarding the use of contact precautions for MRSA-colonized patients, may have influenced findings. Specifically, the recent shift away from isolating MRSA-colonized patients outside of ICUs could have impacted MRSA transmission dynamics within facilities. Another limitation is that we did not assess other infection control strategies being used at VA facilities (ie, infection prevention surveillance software, active surveillance for specific populations), which may have influenced the days of VABA usage and its impact. However, VABA may serve as a primary or supplemental tool for MDRO surveillance.

This study found a significant association between decreased MRSA and CP-CRE HAI rates and the number of days VABA was used. Moving forward, further research is warranted to explore the broader impact of VABA implementation and its effectiveness in diverse healthcare settings. Additionally, ongoing evaluation and adaptation of infection control strategies are essential to address evolving challenges and improve patient outcomes.

## Table 1. Long description

The table presents data on 108 VA facilities categorized by complexity level: low, medium, and high. It includes information on rurality, US region, days of VABA use, facility infection rates for CP-CRE and MRSA, and the number of inpatients for both CP-CRE and MRSA. The table has 108 rows and 8 columns. Column headers are Characteristic, Low complexity N 13, Medium complexity N 16, High complexity N 79, and P-value. Row labels include Rurality, US Region, Days of VABA use, Facility infection rate (CP-CRE), Inpatients (CP-CRE), Facility infection rate (MRSA), and Inpatients (MRSA). Notable trends include a higher concentration of high-complexity facilities in urban areas and varying infection rates and inpatient numbers across different complexity levels.

Navigate back to Table 1..
